# Tinnitus is not associated with cardiovascular risk factors or mortality in the Gutenberg Health Study

**DOI:** 10.1007/s00392-025-02601-y

**Published:** 2025-02-19

**Authors:** Omar Hahad, Berit Hackenberg, Julia Döge, Katharina Bahr-Hamm, Jasmin Ghaemi Kerahrodi, Oliver Tüscher, Matthias Michal, Konstantin Kontohow-Beckers, Alexander K. Schuster, Irene Schmidtmann, Karl J. Lackner, Jörn M. Schattenberg, Stavros Konstantinides, Philipp S. Wild, Thomas Münzel

**Affiliations:** 1 Department of Cardiology – Cardiology I, University Medical Center of the Johannes Gutenberg-University Mainz, Mainz, Germany; 2 German Center for Cardiovascular Research (DZHK), Partner Site Rhine-Main, Mainz, Germany; 3 Department of Otorhinolaryngology, University Medical Center of the Johannes Gutenberg-University Mainz, Mainz, Germany; 4 Department of Psychosomatic Medicine and Psychotherapy, University Medical Center of the Johannes Gutenberg-University Mainz, Mainz, Germany; 5 Department of Psychiatry and Psychotherapy, University Medical Center of the Johannes Gutenberg-University Mainz, Mainz, Germany; 6 Leibniz Institute for Resilience Research (LIR), Mainz, Germany; 7 Preventive Cardiology and Preventive Medicine, Department of Cardiology, University Medical Center of the Johannes Gutenberg-University Mainz, Mainz, Germany; 8 Department of Ophthalmology, University Medical Center of the Johannes Gutenberg-University Mainz, Mainz, Germany; 9 Institute of Medical Biostatistics, Epidemiology & Informatics, University Medical Center of the Johannes Gutenberg-University Mainz, Mainz, Germany; 10 Institute of Clinical Chemistry and Laboratory Medicine, University Medical Center of the Johannes Gutenberg-University Mainz, Mainz, Germany; 11 Metabolic Liver Research Program, I. Department of Medicine, University Medical Center of the Johannes Gutenberg-University Mainz, Mainz, Germany; 12 Center for Thrombosis and Hemostasis (CTH), University Medical Center of the Johannes Gutenberg-University Mainz, Mainz, Germany; 13 Institute for Molecular Biology, Mainz, Germany

**Keywords:** Tinnitus, Tinnitus distress, Cardiovascular disease, Cardiovascular risk factors, Mortality, General population

## Abstract

**Background and aims:**

Tinnitus, characterized by the conscious perception of sound without external acoustic stimulation, presents a multifaceted challenge. Recent research suggests a potential association between tinnitus and cardiovascular health. To elucidate these associations further, we examined the prevalence of tinnitus alongside its distress levels and their associations with cardiovascular risk factors, diseases, and risk of death within a general population cohort.

**Methods and results:**

This study analyzed data from the prospective Gutenberg Health Study (GHS), a population-based cohort of 15,010 individuals aged 35–74, who underwent baseline assessments from 2007 to 2012. We focused on the 10-year follow-up (2017–2020) of the GHS, including otologic testing with 8539 subjects, of whom 2387 (28%) reported tinnitus, allowing for a comprehensive cross-sectional and prospective analysis. Participants completed a questionnaire on hearing-related symptoms, including tinnitus presence (“Do you suffer from ringing in the ears (tinnitus)?” yes/no) and distress (“How much do you feel bothered by it?”), rated on a six-point scale from 0 (“not bothersome”) to 5 (“very bothersome”). Outcomes were assessed based on observed prevalent cardiovascular conditions (i.e., cardiovascular risk factors and diseases) and deaths. Additionally, calculated cardiovascular risk was assessed using the SCORE2 algorithm. Significant differences in baseline characteristics emerged between participants with and without tinnitus, with the former exhibiting advanced age, male predominance, and a higher prevalence of cardiovascular risk factors and diseases. Tinnitus displayed associations with various prevalent cardiovascular diseases including atrial fibrillation (odds ratio 1.48, 95% confidence interval 1.11–1.96), peripheral artery disease (1.43, 1.05–1.95), coronary artery disease (1.49, 1.09–2.04), and any cardiovascular disease (1.31, 1.11–1.56), persisting even after adjustments for demographic, socioeconomic, and cardiovascular risk factors. While crude associations with several prevalent cardiovascular risk factors were observed, these associations diminished upon comprehensive adjustment. Tinnitus presence was associated with elevated 10-year cardiovascular disease risk (incidence rate ratio 1.11, 1.09–1.13), as indicated by higher SCORE 2 values, yet did not predict all-cause mortality risk.

**Conclusions:**

In the present study, tinnitus was associated with prevalent cardiovascular disease. However, no association with cardiovascular risk factors and mortality was found.

## Introduction

Tinnitus, characterized by the perception of sound without external stimuli, varies in intensity and impact among individuals [3]. The underlying mechanisms of tinnitus are complex and influenced by multiple factors [18, 24]. Diagnosis involves assessing its characteristics and impact on quality of life [41]. It is crucial for effective treatment development and understanding of pathophysiological mechanisms to differentiate between the physiological processes causing tinnitus and an individual's response to it [19], including distress and maladaptive behaviors such as social isolation, sleep deprivation, mental health problems, and attempted suicide [8, 14].

Importantly, recent studies suggest a potential association between tinnitus, tinnitus-distress, and cardiovascular health [10, 32, 34, 40]. Tinnitus-related distress, encompassing emotional and cognitive aspects, has been linked to increased cardiovascular risk, possibly through inflammatory and oxidative stress pathways [27, 31, 34, 36, 37]. Chronic stress associated with tinnitus may contribute to systemic inflammation and endothelial dysfunction, both precursors to cardiovascular pathology [27, 34]. Shared mechanisms such as neuroinflammation warrant further exploration [37]. Further, large-scale studies have explored associations between tinnitus and various cardiovascular conditions, revealing links to hypertension [34, 39], hypotension [22], transient ischemic attacks [22], and ischemic cerebrovascular disease [20].

It is important to acknowledge that there may be shared pathomechanisms between tinnitus and cardiovascular disease. However, there is also evidence suggesting that tinnitus manifestation may precede the development of incident events and thus may contribute to an increased risk. Using nationwide data from Taiwan's National Health Insurance Research Database, Huang et al. identified 3474 patients aged 20–45 year diagnosed with incident ischemic cerebrovascular disease, along with 17,370 controls matched for age, sex, and index date [20]. The results revealed a significant association between tinnitus and an increased risk of incident ischemic cerebrovascular disease among young patients, even after adjusting for sex, age, and comorbidities. In conclusion, the authors suggested that tinnitus could potentially serve as a novel risk factor for ischemic stroke in young patients. In support of this conclusion, a recent study from the UK Biobank (N = 140,146) demonstrated a significant association of tinnitus with heightened rates of incident cardiovascular events (hazard ratio (HR) 1.057, 95% confidence interval (CI) 1.017–1.099), myocardial infarction (HR 1.139, 95% CI 1.061–1.222), and all-cause mortality (HR 1.053, 95% CI 1.003–1.105) [42].

A better understanding of the relationship between tinnitus, distress, and cardiovascular risk may inform comprehensive strategies for management. Addressing tinnitus and distress could emerge as a potential strategy for cardiovascular risk reduction, considering shared mechanisms and traditional cardiovascular risk factors exhibited by individuals with tinnitus. Therefore, the aim of the present study was to comprehensively examine the association between tinnitus, cardiovascular risks, and risk of death using data from a large cohort of the general population.

## Methods

### The Gutenberg health study—study design and sample

We analyzed data from the Gutenberg Health Study (GHS), which involved 15,010 individuals aged 35–74 years (referred to as the core cohort) who underwent a thorough 5-h baseline examination between 2007 and 2012 at the University Medical Center Mainz, Germany [15, 17, 38]. These examinations followed standardized protocols, including various interviews and clinical tests. Follow-up exams occurred at 5 and 10-year intervals after enrollment, from 2012 to 2017 and from 2017 to 2022, respectively. Otologic testing was added to the study at the 10-year follow-up (2017–2020). Additionally, new participants were recruited: a young cohort aged 25–44 years (n = 4000) and a senior cohort aged 75–85 years (n = 1000). All three cohorts will continue to be followed up every 10 years until 2027 [14]. The GHS procedures were approved by the ethics committee of the Statutory Physician Board of the State Rhineland-Palatinate (reference number 837.020.07(5555)) and adhered to ethical guidelines outlined in the Declaration of Helsinki. Prior to participation, each individual provided written consent.

### Tinnitus presence and distress

During a structured interview, participants completed a questionnaire regarding their hearing-related symptoms, which included standardized items about the presence and distress caused by tinnitus [14]. Participants were asked, “Do you suffer from ringing in the ears?” with options for a yes or no response. Additionally, they were asked, “How much do you feel bothered by it?” with a six-point scale ranging from 0 ("not bothersome") to 5 ("very bothersome").

### Cardiovascular disease, risk factors, and deaths

Prevalent (cardiovascular) disease was determined by reviewing medical records containing physician diagnoses or diagnoses made during study visits. It encompassed several conditions, including atrial fibrillation, coronary artery disease, myocardial infarction, stroke, chronic heart failure, peripheral artery disease, and chronic kidney disease. To update mortality records, we conducted quarterly inquiries to both the registry offices and the mortality registry in Rhineland-Palatinate. Official death certificates were obtained for a thorough review. Socioeconomic status was evaluated using a validated scale that considers educational background, current occupation, and income, ranging from 3 to 21 [23]. A higher score indicates a higher socioeconomic status. Regarding smoking status, participants were categorized as either current smokers or non-smokers. Current smokers were defined as those who smoked regularly or daily, consuming at least one cigarette per day, seven per week, or one pack per month for at least the past six months. Non-smokers included never smokers, former smokers who had quit for longer than six months, and occasional smokers. Diabetes mellitus was identified by a physician's diagnosis, use of antidiabetic medication, fasting blood glucose levels of 126 mg/dL or higher after an overnight fast, non-fasting blood glucose levels of 200 mg/dL or higher, or an HbA1c level of 6.5% or higher. Arterial hypertension was diagnosed if individuals were taking antihypertensive medication or had a resting systolic blood pressure of 140 mmHg or higher or a diastolic blood pressure of 90 mmHg or higher (averaged from the second and third readings after 8 and 11 min of rest). Obesity was defined as having a body mass index of 30 kg/m^2^ or higher [2]. The cut-off for non-HDL cholesterol was > 130 mg/dL, based on [33]. A family history of myocardial infarction or stroke was noted if a first-degree relative (female ≤ 65 years or male ≤ 60 years) had experienced such an event. To differentiate between seemingly healthy individuals in Europe with low and high cardiovascular disease risk, a new tool called SCORE2 (Systematic COronary Risk Evaluation) has been developed [12]. This tool predicts the 10-year risk of experiencing fatal and non-fatal cardiovascular events. SCORE2 is built upon extensive data from nearly 700,000 patients across various large prospective studies and has been validated externally in over one million individuals. The risk models in SCORE2 are adjusted to account for the background cardiovascular disease risk at the country level, using standardized cardiovascular disease mortality rates, resulting in four categories of country-wide cardiovascular disease risk: low, moderate, high, and very high. These prediction models in Europe are based on traditional cardiovascular risk factors such as age, sex, smoking status, diabetes, blood pressure, and cholesterol levels [12, 21].

### Statistical analysis

Participants with incomplete data on tinnitus presence were excluded from the analysis. Baseline continuous variables are shown as mean and standard deviation and tested with T-test or if skewness > 1 by median (Q1, Q3) and tested with U-Test. Baseline binary variables are described as relative and absolute frequencies and tested with chi-squared test. Binary logistic regression analysis was conducted to determine the association between the presence of tinnitus and tinnitus distress in subjects with prevalent tinnitus (independent variables) and prevalent cardiovascular disease and cardiovascular risk factors (dependent variables), with odds ratios (OR), 95% CI, and *P* values reported. Incidence rate ratios (IRR) and their corresponding 95% CI were computed through Poisson regression models to investigate the relationship between the presence of tinnitus and tinnitus distress in subjects with prevalent tinnitus (independent variables) and SCORE 2 (dependent variable). Furthermore, HR and their corresponding 95% CI were determined using Cox regression models to explore the association between the presence of tinnitus or tinnitus distress in subjects with prevalent tinnitus (independent variables) and the risk of all-cause death (dependent variable). Kaplan–Meier analysis was employed to assess the relationship between the presence of tinnitus and tinnitus distress in subjects with prevalent tinnitus and the risk of all-cause death, with *P* values obtained from the log-rank test. Sequential adjustments were made in the models: Model 1 was crude. Model 2 was additionally adjusted for sex, age, and socioeconomic status. Model 3 was additionally adjusted for current smoking, diabetes mellitus, arterial hypertension, obesity, non-HDL cholesterol, family history of myocardial infarction or stroke, and chronic kidney disease (specific adjustments can be found in the table legends). *P* values were interpreted as continuous measures of evidence against the null hypothesis and were therefore reported precisely. *P* values less than 0.05 were considered indicative of significant associations for descriptive purposes. Statistical analyses were conducted using the R software package (http://www.r-project.org/).

## Results

### Baseline characteristics

Baseline characteristics of the study sample (*N* = 8539), stratified by tinnitus presence, revealed significant differences (Table 1). Participants with tinnitus (n = 2387) were more likely to be older and male. They also exhibited a higher prevalence of cardiovascular risk factors including diabetes mellitus, arterial hypertension, and (cardiovascular) diseases including atrial fibrillation, coronary artery disease, myocardial infarction, stroke, chronic heart failure, peripheral artery disease, and chronic kidney disease. Tinnitus participants had a higher median SCORE 2 value, indicating greater 10-year cardiovascular disease risk. Tinnitus distress levels varied, with 24.9% experiencing no distress and 2.8% experiencing severe distress.

**Table 1:** Baseline characteristics (2017–2020) of the Gutenberg Health Study sample stratified by the presence of tinnitus (*N* = 8,539)

	No present tinnitus (n = 6152)	Present tinnitus (n = 2387)	*P*-value
Women–% (no.)	51.6 (3172)	42.1 (1005)	** < 0.0001**
Age–years	60.0 (14.0)	62.2 (12.8)	** < 0.0001**
Socioeconomic status (SES)–score	14.14 (4.23)	13.99 (4.23)	0.13
Current smoking–% (no.)	13.0 (786)	13.0 (304)	0.97
Diabetes mellitus–% (no.)	10.0 (615)	12.6 (298)	**0.00090**
Arterial hypertension–% (no.)	51.6 (3170)	56.9 (1358)	** < 0.0001**
Obesity–% (no.)	25.9 (1586)	28.5 (676)	**0.019**
Non-HDL cholesterol > 130 mg/dL–% (no.)	70.3 (4310)	71.6 (1697)	0.26
Family history of myocardial infarction or stroke–% (no.)	3.3 (200)	4.3 (102)	**0.026**
Myocardial infarction–% (no.)	1.1 (70)	1.9 (46)	**0.0065**
Stroke–% (no.)	1.3 (82)	2.0 (48)	**0.023**
Atrial fibrillation–% (no.)	2.5 (156)	4.1 (97)	**0.00027**
Peripheral artery disease–% (no.)	2.1 (129)	3.5 (83)	**0.00033**
Coronary artery disease–% (no.)	2.0 (124)	3.6 (85)	** < 0.0001**
Chronic heart failure–% (no.)	1.7 (101)	2.7 (63)	**0.0036**
Any cardiovascular disease–% (no.)	8.8 (535)	12.8 (303)	** < 0.0001**
Chronic kidney disease–% (no.)	3.8 (230)	5.9 (140)	** < 0.0001**
SCORE 2	5.00 (2.00/10.00)	6.00 (3.00/11.00)	** < 0.0001**
SCORE 2 (in individuals without cardiovascular disease)	4.00 (2.00/9.00)	6.00 (3.00/10.00)	** < 0.0001**
Tinnitus distress			
0–% (no.)	–	24.9 (587)	–
1–% (no.)	–	33.0 (777)	–
2–% (no.)	–	19.2 (451)	–
3–% (no.)	–	13.6 (321)	–
4–% (no.)	–	6.5 (152)	–
5–% (no.)	–	2.8 (67)	–

Continuous variables are shown as mean and standard deviation and tested with T-test or if skewness > 1 by median (Q1, Q3) and tested with U-Test. Binary variables are described as relative and absolute frequencies and tested with chi-squared test

Socioeconomic status score ranges from 3 to 21 with higher values indicating higher status

SCORE 2 denotes Systematic COronary Risk Evaluation

### Tinnitus (distress) and prevalent cardiovascular disease

Tinnitus showed significant positive associations with several cardiovascular diseases (Table 2). In the crude model (Model 1), tinnitus was significantly associated with increased odds of myocardial infarction, stroke, atrial fibrillation, peripheral artery disease, coronary artery disease, chronic heart failure, and any cardiovascular disease (composite variable). After adjusting for sex, age, and socioeconomic status (Model 2), these associations remained significant except for myocardial infarction and stroke. Further adjustments in Model 3, including additional cardiovascular risk factors, maintained significant associations with atrial fibrillation, peripheral artery disease, coronary artery disease, and any cardiovascular disease.

**Table 2:** Odds ratios (OR) and 95% confidence intervals (CI) were calculated using binary logistic regression models to examine the association between the presence of tinnitus (independent variable) and prevalent cardiovascular disease (dependent variable) in the Gutenberg Health Study (2017–2020)

Tinnitus	*N*/events	Model 1		Model 2		Model 3	
		OR [95% CI]	*P* value	OR [95% CI]	*P* value	OR [95% CI]	*P* value
Myocardial infarction	7990/97	1.71 [1.17; 2.49]	**0.0052**	1.49 [0.99; 2.22]	0.054	1.36 [0.89; 2.809]	0.16
Stroke	8006/113	1.53 [1.07; 2.19]	**0.021**	1.35 [0.93; 1.96]	0.12	1.31 [0.88; 1.94]	0.18
Atrial fibrillation	7991/223	1.63 [1.26; 2.11]	**0.00021**	1.48 [1.12; 1.94]	**0.0052**	1.48 [1.11; 1.96]	**0.0076**
Peripheral artery disease	7929/182	1.70 [1.28; 2.24]	**0.00022**	1.49 [1.11; 2.01]	**0.0082**	1.43 [1.05; 1.95]	**0.023**
Coronary artery disease	7934/182	1.90 [1.36; 2.38]	** < 0.0001**	1.55 [1.16; 2.09]	**0.0034**	1.49 [1.09; 2.04]	**0.013**
Chronic heart failure	7925/147	1.63 [1.19; 2.24]	**0.0026**	1.48 [1.07; 2.06]	**0.019**	1.32 [0.93; 1.86]	0.12
Any cardiovascular disease	7964/740	1.53 [1.32; 1.77]	** < 0.0001**	1.35 [1.15; 1.58]	**0.00032**	1.31 [1.11; 1.56]	**0.0019**

*N*/events denotes model 3

Model 1 was crude

Model 2 was additionally adjusted for sex, age, and socioeconomic status

Model 3 was additionally adjusted for current smoking, diabetes mellitus, arterial hypertension, obesity, non-HDL cholesterol, family history of myocardial infarction or stroke, and chronic kidney disease

In Model 1, significant associations were found between tinnitus distress and peripheral artery disease, coronary artery disease, and any cardiovascular disease (Table 3). In Model 3, a significant association persisted with coronary artery disease (OR 1.20 per point increase in tinnitus distress).

**Table 3:** Odds ratios (OR) and 95% confidence intervals (CI) were calculated using binary logistic regression models to examine the association between tinnitus distress in subjects with prevalent tinnitus (independent variable) and prevalent cardiovascular disease (dependent variable) in the Gutenberg Health Study (2017–2020)

Tinnitus distress	*N*/events	Model 1		Model 2		Model 3	
		OR per point increase [95% CI]	*P* value	OR per point increase [95% CI]	*P* value	OR per point increase [95% CI]	*P* value
Myocardial infarction	2398/40	1.14 [0.94; 1.39]	0.19	1.12 [0.91; 1.38]	0.28	1.12 [0.89; 1.41]	0.34
Stroke	2399/51	1.10 [0.91; 1.33]	0.31	1.05 [0.87; 1.27]	0.61	1.05 [0.86; 1.29]	0.64
Atrial fibrillation	2397/96	1.04 [0.90; 1.20]	0.57	1.03 [0.89; 1.19]	0.70	1.03 [0.89; 1.21]	0.67
Peripheral artery disease	2372/77	1.21 [1.05; 1.40]	**0.0085**	1.18 [1.02; 1.38]	**0.028**	1.15 [0.98; 1.34]	0.095
Coronary artery disease	2380/77	1.22 [1.05; 1.41]	**0.0086**	1.19 [1.03; 1.39]	**0.023**	1.20 [1.02; 1.41]	**0.032**
Chronic heart failure	2370/60	0.99 [0.82; 1.18]	0.89	0.99 [0.83; 1.19]	0.92	0.95 [0.782; 1.16]	0.62
Any cardiovascular disease	2391/296	1.11 [1.02; 1.21]	**0.013**	1.09 [0.99; 1.19]	0.060	1.08 [0.98; 1.18]	0.13

*N*/events denotes model 3

Model 1 was crude

Model 2 was additionally adjusted for sex, age, and socioeconomic status

Model 3 was additionally adjusted for current smoking, diabetes mellitus, arterial hypertension, obesity, non-HDL cholesterol, family history of myocardial infarction or stroke, and chronic kidney disease

### Tinnitus (distress) and prevalent cardiovascular risk factors

Tinnitus was initially (Model 1) associated with arterial hypertension, obesity, and diabetes mellitus (Table 4). However, after full adjustment (Model 3), none of these associations remained significant.

**Table 4:** Odds ratios (OR) and 95% confidence intervals (CI) were calculated using binary logistic regression models to examine the association between the presence of tinnitus (independent variable) and prevalent cardiovascular risk factors (dependent variable) in the Gutenberg Health Study (2017–2020)

Tinnitus	*N*/events	Model 1		Model 2		Model 3	
		OR [95% CI]	*P* value	OR [95% CI]	*P* value	OR [95% CI]	*P* value
Arterial hypertension	8016/4231	1.24 [1.13; 1.36]	** < 0.0001**	1.06 [0.95; 1.18]	0.30	1.05 [0.94; 1.18]	0.37
Non-HDL cholesterol	8016/5678	1.06 [0.96; 1.18]	0.26	1.06 [0.95; 1.18]	0.27	1.07 [0.95; 1.19]	0.26
Obesity	8016/2116	1.13 [1.07; 1.26]	**0.023**	1.033 [0.93; 1.16]	0.56	0.99 [0.89; 1.12]	0.93
Diabetes mellitus	8016/830	1.29 [1.11; 1.49]	**0.00079**	1.09 [0.93; 1.28]	0.27	1.05 [0.89; 1.24]	0.54

*N*/events denotes model 3

Model 1 was crude

Model 2 was additionally adjusted for sex, age, and socioeconomic status

Model 3 was additionally adjusted for current smoking, diabetes mellitus, arterial hypertension, obesity, non-HDL cholesterol, family history of myocardial infarction or stroke, and chronic kidney disease (except for the specific variable that constituted the dependent variable in this context)

No significant associations were found between tinnitus distress and cardiovascular risk factors after full adjustment (Model 3, Table 5).

**Table 5:** Odds ratios (OR) and 95% confidence intervals (CI) were calculated using binary logistic regression models to examine the association between tinnitus distress in subjects with prevalent tinnitus (independent variable) and prevalent cardiovascular risk factors (dependent variable) in the Gutenberg Health Study (2017–2020)

Tinnitus distress	*N*/events	Model 1		Model 2		Model 3	
		OR per point increase [95% CI]	*P* value	OR per point increase [95% CI]	*P* value	OR per point increase [95% CI]	*P* value
Arterial hypertension	2405/1396	1.04 [0.98; 1.11]	0.15	1.03 [0.97; 1.10]	0.36	1.02 [0.95; 1.10]	0.60
Non-HDL cholesterol	2405/1740	0.99 [0.93; 1.06]	0.82	1.00 [0.94; 1.07]	0.99	1.01 [0.94; 1.08]	0.89
Obesity	2405/668	1.08 [1.02; 1.15]	**0.015**	1.06 [0.99; 1.13]	0.074	1.051 [0.98; 1.13]	0.16
Diabetes mellitus	2405/289	1.08 [0.99; 1.17]	0.093	1.03 [0.94; 1.12]	0.56	1.03 [0.93; 1.13]	0.61

*N*/events denotes model 3

Model 1 was crude

Model 2 was additionally adjusted for sex, age, and socioeconomic status

Model 3 was additionally adjusted for current smoking, diabetes mellitus, arterial hypertension, obesity, non-HDL cholesterol, family history of myocardial infarction or stroke, and chronic kidney disease (except for the specific variable that constituted the dependent variable in this context)

### Tinnitus (distress), SCORE 2, and risk of all-cause death

The presence of tinnitus showed a significant positive association with SCORE 2 across all models, indicating an increased 10-year cardiovascular disease risk (Table 6). However, a significant inverse association was found between tinnitus distress and SCORE 2 across Model 2 and 3.

**Table 6:** Incidence rate ratios (IRR) and 95% confidence intervals (CI) were calculated using Poisson regression models to examine the association between presence of tinnitus or tinnitus distress in subjects with prevalent tinnitus (independent variables) and SCORE 2 (dependent variable) in the Gutenberg Health Study (2017–2020)

SCORE 2	*N*	Model 1		Model 2		Model 3	
		IRR [95% CI]	*P* value	IRR [95% CI]	*P* value	IRR [95% CI]	*P* value
Presence of tinnitus	7220	1.12 [1.10; 1.14]	** < 0.0001**	1.12 [1.10; 1.14]	** < 0.0001**	1.11 [1.09; 1.13]	** < 0.0001**
Tinnitus distress (per point increase)	2094	0.99 [0.99; 1.01]	0.63	0.99 [0.98; 0.99]	**0.0094**	0.98 [0.97; 0.99]	**0.00032**

*N* denotes model 3

Model 1 was crude

Model 2 was additionally adjusted for socioeconomic status

Model 3 was additionally adjusted for obesity, family history of myocardial infarction or stroke, and chronic kidney disease

**Fig. 1:**
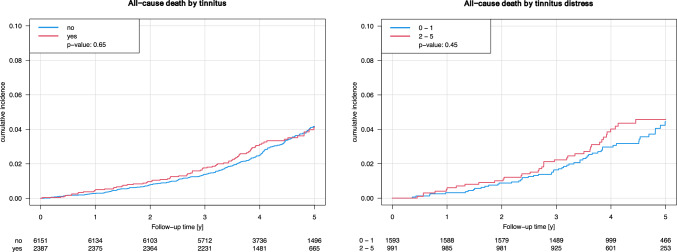
Kaplan–Meier curves illustrating the association between the presence of tinnitus (left graph) and tinnitus distress (0–1 vs. 2–5, right graph) with risk of all-cause death in the Gutenberg Health Study (2017–2022). The *P* value corresponds to the log-rank test

Neither the presence of tinnitus nor tinnitus distress showed a significant association with the risk of all-cause death in any of the models (Table 7 and Fig. 1).

**Table 7:** Hazard ratios (HR) and 95% confidence intervals (CI) were calculated using Cox regression models to examine the association between presence of tinnitus or tinnitus distress in subjects with prevalent tinnitus (independent variables) and risk of all-cause death (dependent variable) in the Gutenberg Health Study (2017–2024)

All-cause death	*N*	Model 1		Model 2		Model 3	
		HR [95% CI]	*P* value	HR [95% CI]	*P* value	HR [95% CI]	*P* value
Presence of tinnitus	8015	1.06 [0.82; 1.39]	0.65	0.92 [0.70; 1.21]	0.54	0.96 [0.72; 1.27]	0.75
Tinnitus distress (per point increase)	2405	0.97 [0.83; 1.14]	0.73	0.96 [0.83; 1.13]	0.64	0.97 [0.82; 1.14]	0.68

*N* denotes model 3

Model 1 was crude

Model 2 was additionally adjusted for sex, age, and socioeconomic status

Model 3 was additionally adjusted for current smoking, diabetes mellitus, arterial hypertension, obesity, non-HDL cholesterol, family history of myocardial infarction or stroke, and chronic kidney disease

In the no tinnitus group, 5,968 individuals did not experience the event, while 184 individuals did. In the tinnitus group, 2,310 individuals did not experience the event, and 77 individuals did. The counts for individuals experiencing no event across the tinnitus distress levels 0 to 5 are 777, 763, 432, 311, 146, and 64, respectively. For individuals experiencing the event, the corresponding counts are 39, 14, 19, 10, 6, and 3

## Discussion

The findings of our study shed light on the potential relationship between tinnitus and related distress, cardiovascular risks, and risk of death within a large cohort of individuals from the general population. Our analysis revealed several notable findings. Individuals reporting tinnitus exhibited a significantly higher prevalence of cardiovascular risk factors, including arterial hypertension, obesity, diabetes mellitus, and family history of myocardial infarction or stroke, compared to those without tinnitus. Furthermore, our study identified significant associations between tinnitus and cardiovascular diseases, such as atrial fibrillation, peripheral artery disease, and coronary artery disease. Importantly, these associations persisted even after adjusting for relevant demographic, socioeconomic, and cardiovascular risk factors. While a general trend suggested an association between tinnitus distress and cardiovascular diseases, notably coronary artery disease, these associations attenuated following full adjustment. Moreover, our study demonstrated a positive association between the presence of tinnitus and the SCORE 2, a validated tool for predicting the 10-year risk of cardiovascular events in seemingly healthy individuals, while a significant inverse, but statistically negligible, association with tinnitus distress was found. This association remained significant across all models, suggesting that individuals with tinnitus may face an elevated cardiovascular disease risk over time. However, it is important to note that our study did not find significant associations between tinnitus, either presence or distress, and the risk of all-cause death. Likewise, while tinnitus initially showed associations with certain cardiovascular risk factors, these associations diminished overall after thorough adjustment. Also, tinnitus distress did not exhibit significant associations with cardiovascular risk factors following comprehensive adjustment.

The relationship between tinnitus and cardiovascular health has been a subject of growing interest. While the exact mechanisms underlying tinnitus remain elusive, recent research suggests that its manifestation and progression may be influenced by a myriad of factors, including neurophysiological changes, psychological distress, and systemic health conditions [3]. Of particular interest is the emerging evidence pointing towards a bidirectional relationship between tinnitus and cardiovascular health [4]. Several studies have highlighted the potential role of chronic stress and inflammation in linking these two seemingly distinct conditions [34]. It is hypothesized that the persistent distress associated with tinnitus may trigger physiological responses, such as increased sympathetic activity and elevated levels of inflammatory markers, which in turn could contribute not only to the development or exacerbation of mental health conditions [29] but also to cardiovascular diseases. The bidirectional relationship between mental and cardiovascular health is well-established [28]. Conversely, individuals with pre-existing cardiovascular conditions may experience heightened susceptibility to tinnitus due to compromised vascular function [5, 27] or altered neural signaling pathways [35]. Moreover, studies have suggested links between tinnitus and specific cardiovascular diseases [10, 32, 34, 40], suggesting potential shared pathophysiological pathways. Conditions such as hypertension [34, 39], ischemic stroke [20, 22], and atherosclerosis [40] have been reported to co-occur with or to follow tinnitus. Further pathways may include tinnitus-associated sleep disturbance and behavioral responses to tinnitus-distress. Tinnitus can interfere with sleep, resulting in sleep disturbances such as insomnia or fragmented sleep patterns [11, 13]. Poor sleep quality per se is associated with an increased risk of cardiovascular diseases including hypertension, coronary artery disease, and stroke [7, 26]. Individuals experiencing tinnitus distress may have higher risk to adopt and maintain unhealthy behaviors and coping strategies such as smoking, excessive alcohol consumption, poor dietary habits, and physical inactivity, all of which increase the risk of cardiovascular diseases [6, 9, 25]. Tinnitus distress may furthermore impact healthcare seeking and adherence to medical treatments as recent studies in individuals suffering from stress suggest [1, 30]. All these factors may also contribute to an elevated risk of death in patients with tinnitus. These findings may not only underscore the importance of considering tinnitus as a potential marker for underlying cardiovascular pathology but also raise questions about the temporal sequence of events and potential causal relationships between these conditions.

The current study has notable strengths as well as potential limitations. Among its strengths is the use of a dataset derived from a large, population-representative sample from Germany, enabling the comprehensive investigation of various cardiovascular risk factors, diseases, and death risks. However, it is essential to acknowledge certain limitations. Primarily, the observational and partly cross-sectional design of the study precludes making causal inferences or precisely determining the direction of effects. Nevertheless, it is worth noting that evidence of a bidirectional association does not necessarily invalidate causal explanations in either or both directions of an association [16]. Assessment of tinnitus and distress relied solely on single-item personal interviews, thereby relying on individual compliance. Consequently, comparability with previous research is limited due to the heterogeneity in tinnitus assessment. Specifically, our questionnaire did not incorporate tinnitus duration or distinguish between intermittent and continuous symptoms. Additionally, the tinnitus definition used in our study captures the participants current tinnitus status, reflecting the point prevalence at the time of assessment rather than the lifetime occurrence. Thus, we cannot differentiate between acute tinnitus, chronic tinnitus, and history of any tinnitus. We only found limited evidence for an association between tinnitus distress and all outcomes of interest, which largely contrasts with previous results. This discrepancy may be attributed to differing definitions of predictors and outcomes or limited statistical power due to a smaller sample size. We acknowledged that residual confounding may have influenced our findings as some underlying factors affecting both tinnitus and cardiovascular outcomes may have remained unmeasured despite our adjustments. This highlights the need for future studies to further elucidate the pathophysiological mechanisms linking tinnitus with cardiovascular disease.

In conclusion, our findings contribute to a deeper understanding of the complex relationship between tinnitus and cardiovascular health within a population-based cohort. While further research is warranted to elucidate the underlying mechanisms and potential causality of these associations, our study may suggest the importance of considering tinnitus as a potential marker for cardiovascular risk assessment. Future studies should explore targeted interventions aimed at managing tinnitus and its associated distress as part of comprehensive strategies for cardiovascular risk reduction.

## Data Availability

The analysis presents clinical data of a large-scale population-based cohort with ongoing follow-up examinations. This project constitutes a major scientific effort with high methodological standards and detailed guidelines for analysis and publication to ensure scientific analyses on the highest level. Therefore, data are not made available for the scientific community outside the established and controlled workflows and algorithms. To meet the general idea of verification and reproducibility of scientific findings, we offer access to data at the local database in accordance with the ethics vote on request at any time. The GHS steering committee, which comprises a member of each involved department and the head of the GHS, convenes once a month. The steering committee decides on internal and external access of researchers and use of the data and biomaterials based on a research proposal to be supplied by the researcher. Interested researchers make their requests to the head of the GHS (Philipp S. Wild, philipp.wild@unimedizin-mainz.de).
